# The Effect of Acetylsalicylic Acid, as a Representative Non-Steroidal Anti-Inflammatory Drug, on the Activity of Myeloperoxidase

**DOI:** 10.3390/ph16071012

**Published:** 2023-07-17

**Authors:** Beata Gąsowska-Bajger, Kinga Sosnowska, Agnieszka Gąsowska-Bodnar, Lubomir Bodnar

**Affiliations:** 1 Institute of Chemistry, Opole University, 45-052 Opole, Poland; k.sosnowska_98@o2.pl; 2 Faculty of Medical and Health Sciences, Siedlce University of Natural Sciences and Humanities, 08-110 Siedlce, Poland; agnga@mp.pl (A.G.-B.); lubo@esculap.pl (L.B.)

**Keywords:** myeloperoxidase, non-steroidal anti-inflammatory drugs, acetylsalicylic acid

## Abstract

Background: Acetylsalicylic acid (ASA or aspirin) is one of the world’s most widely used non-steroidal anti-inflammatory drug (NSAID). Numerous studies have shown that the long-term use of aspirin may contribute to longer survival among patients with various types of cancer, including ovarian cancer. Aim: The aim of this study was to investigate the effect of ASA on myeloperoxidase (MPO), which is found at an elevated level in women with ovarian cancer, among others. Methods: The influence of different concentrations of ASA on the chlorinating and peroxidase activity of MPO was analysed. The relationship between the concentration of ASA and the degree of inhibition of MPO activity was determined based on the results. Conclusions: Aspirin has a significant effect on MPO activity. The use of 50 mM ASA resulted in the enzyme activity being inhibited by more than 90%.

## 1. Introduction

Myeloperoxidase (MPO), an enzyme belonging to the peroxidase group, is released in the phagocytosis process and catalyses the oxidation of various types of compounds, such as halides (Cl^−^, Br^−^, I^−^) or thiocyanates (SCN^−^), in the presence of hydrogen peroxide to produce the corresponding acids: hypochlorous acid (HOCl), hypobromous acid (HOBr), hypoiodous acid (HOI) and hypothiocyanous acid (HOSCN), which are strong and effective antimicrobial substances [1]. Whilst the oxidants produced by MPO play an important role in the destruction of microorganisms and the inactivation of viruses, there is growing evidence that the increased activity of this enzyme and the accumulation of peroxidase-induced oxidative damage contribute to the development of many life-threatening diseases, many of which are related to the circulatory system, such as atherosclerosis or acute coronary syndrome. In addition to these diseases, MPO also contributes to obesity, diabetes and diseases of the nervous system, such as depression. However, the processes by which these diseases form are not yet fully understood. According to the latest research, the enzyme also contributes to the formation of various types of cancer, including lung cancer and ovarian cancer [2,3,4,5]. Patients with gynaecological malignancies have also been shown to have elevated plasma MPO levels and tissue expression [6].

Non-steroidal anti-inflammatory drugs (NSAIDs) are one of the most widespread groups of medicinal drugs used around the world. Since they inhibit cyclooxygenase (COX) enzymes, they are used not only as painkillers but also as antipyretics and anti-inflammatory drugs. The COX enzyme participates in the transformation of arachidonic acid. The activation of COX not only releases thromboxane but also prostacyclins and prostaglandins, which participate in the thermoregulation process and are transmitters of pain impulses [7,8]. Since NSAIDs show great diversity in terms of chemical structure and clinical properties, there are five different forms of classification of these compounds, according to certain, specific criteria. Based on their chemical structure, NSAIDS are divided into: salicylates, phenylacetic acid derivatives, phenylpropionic acid derivatives, fenamic acid derivatives, indoleacetic acid derivatives and enolic acid derivatives [7,8].

Acetylsalicylic acid (ASA), as an acetyl derivative of salicylic acid, is a widely used NSAID. Its wide range of action as an anti-inflammatory, antipyretic and analgesic drug is explained by the fact that one of the main mechanisms of ASA action is the inhibition of the synthesis of prostanoids (compounds involved in inflammatory processes) with a wide range of activities in the body [9]. Additionally, aspirin has been found to inhibit various signalling pathways, such as the Ras/c-Raf, extracellular signal-regulated kinase (ERK)/MAPK, NF-B, and mTOR pathways, in various cancer cells. The chemopreventive mechanism of aspirin may also include increasing the number of tumour-infiltrating lymphocytes in the tumour microenvironment [10]. Studies have shown that the long-term use of aspirin reduces the risk of gastrointestinal cancers [7,11]. Of particular interest is its effect on the reduced incidence of colorectal cancer, which is the third most common cancer in the world. In addition, longer survival has also been noted among patients diagnosed with ovarian cancer who regularly take ASA compared to patients who have never used them [12,13].

## 2. Results

### 2.1. Oxidation of ASA by MPO

**Figure 1:**
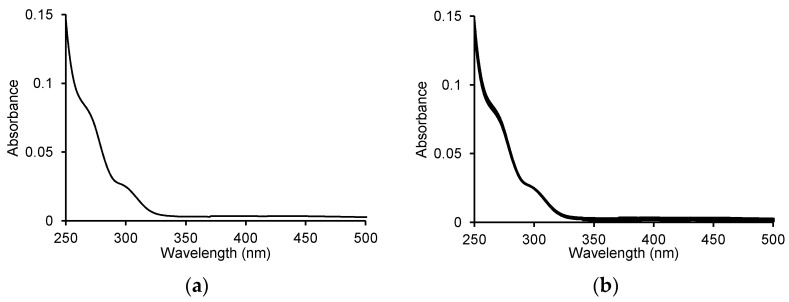
(**a**) UV-Vis spectrum for 100 μM acetylsalicylic acid and (**b**) the spectra recorded during the oxidation of 100 μM ASA by 5 μg/mL (0.25 U/mL) myeloperoxidase in the presence of 100 μM H_2_O_2_ in 50 mM sodium phosphate buffer, pH 7.4 at 25 °C. The spectra were recorded every 1 min for 10 min.

We started our analyses by recording the spectrum of ASA itself and checking the changes after adding the enzyme to the ASA. We did not observe changes in the spectra over time that would indicate that this compound is a substrate for MPO (Figure 1).

### 2.2. Oxidation of O-Dianisidine Dihydrochloride by MPO in the Presence of ASA

**Figure 2:**
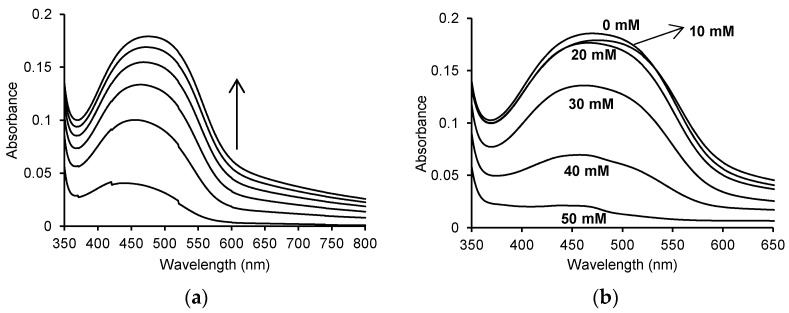
(**a**) Time spectra recorded for the oxidation of 130 μM *o*-dianisidine by 5 μg/mL (0.25 U/mL) myeloperoxidase in the presence of 60 μM H_2_O_2_ in 50 mM sodium phosphate buffer, pH 7.4 at 25 °C; (**b**) spectra after 10 min for the enzymatic oxidation of 130 μM *o*-dianisidine in the presence of 60 μM H_2_O_2_ without ASA and in the presence of 10 mM, 20 mM, 30 mM, 40 mM and 50 mM ASA in 50 mM sodium phosphate buffer, pH 7.4 at 25 °C. The concentration of myeloperoxidase was 5 μg/mL (0.25 U/mL) in all reactions.

One of the two activities exhibited by MPO is peroxidase activity, which is verified by the oxidation of *o*-dianisidine by MPO in the presence of hydrogen peroxide. This reaction produces the orange-brown oxidised form of *o*-dianisidine. The *o*-dianisidine oxidation reaction proceeds over time, and its progress was studied using a Jasco V-650 UV-Vis spectrophotometer. Figure 2a shows the UV-Vis spectra of the reaction in the range of 350 to 800 nm, recorded every 2 min for 10 min.

Recording the spectra of this reaction allowed the identification of the wavelength at which the resulting product showed the highest absorbance. The oxidised form of *o*-dianisidine showed an absorbance maximum at a wavelength of approx. 460 nm, which shifted towards longer wavelengths over time. The addition of various concentrations of ASA to the mixture resulted in a decrease in the absorbance at 460 nm (Figure 2b).

When recording the UV-Vis spectra with and without the addition of ASA in the reaction mixture, visible differences in absorbance were observed. The maximum absorbance at 460 nm was 0.185 ± 0.016% for the enzymatic reaction without the addition of ASA. A decrease in the absorbance value was observed after adding appropriate concentrations of ASA, corresponding to 0.008 for 10 mM ASA (A_460 nm_ = 0.177 ± 0.02%), 0.009 for 20 mM ASA (A_460 nm_ = 0.176 ± 0.01%), 0.049 for 30 mM ASA (A_460 nm_ = 0.136 ± 0.003%), 0.116 for 40 mM ASA (A_460 nm_ = 0.069 ± 0.002%) and 0.164 for 50 mM ASA (A_460 nm_ = 0.021 ± 0.005%).

**Figure 3:**
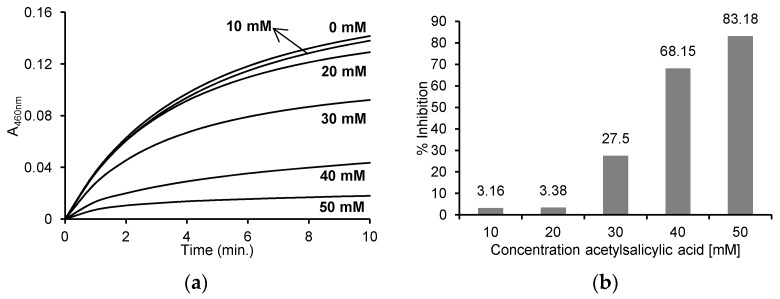
(**a**) Reaction progress curves at 460 nm wavelength for the enzymatic oxidation of 130 μM *o*-dianisidine in the presence of 60 μM H_2_O_2_ without ASA and in the presence of 10 mM, 20 mM, 30 mM, 40 mM and 50 mM ASA in 50 mM sodium phosphate buffer, pH 7.4 at 25 °C; (**b**) the % inhibition of individual concentrations of ASA after the enzymatic oxidation of 130 μM *o*-dianisidine in the presence of 60 μM H_2_O_2_ and after the addition of 10 mM, 20 mM, 30 mM, 40 mM and 50 mM ASA in 50 mM sodium phosphate buffer, pH 7.4 at 25 °C. The concentration of myeloperoxidase was 5 μg/mL (0.25 U/mL) in all reactions.

The initial speed of the individual reactions involving different concentrations of ASA were determined based on the recorded reaction progress curves at 460 nm (Figure 3a), and the % inhibition of the reaction by the appropriate concentration of ASA was determined (Figure 3b). The obtained results show the reaction rate was inhibited by 3.16% ± 0.054 and 3.38 ± 5.16% in the case of 10 mM and 20 mM ASA, respectively. The presence of higher concentrations of ASA (30, 40 and 50 mM) resulted in the significant inhibition of the reaction, by 27.50 ± 4.51%, 68.15 ± 4.93% and 83.18 ± 5.40%, respectively.

### 2.3. Oxidation of MCD by MPO in the Presence of ASA

We used the reaction with MCD to measure the MPO chlorinating activity. This reaction takes place in the presence of chloride ions and hydrogen peroxide, and the product of the reaction is dichlorodimedone. The decrease in absorbance of MCD is recorded at 290 nm.

**Figure 4:**
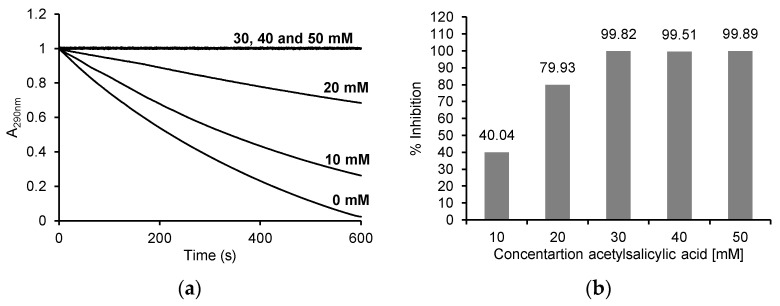
(**a**) Reaction progress curves at 290 nm for the enzymatic oxidation of 50 μM monochlorodimedone in the presence of 100 mM NaCl and 50 μM H_2_O_2_ without ASA and in the presence of 10 mM, 20 mM, 30 mM, 40 mM and 50 mM ASA in 50 mM acetate buffer, pH 5.0 at 25 °C; (**b**) the % inhibition for individual concentrations of ASA after enzymatic oxidation of 50 μM monochlorodimedone in the presence of 100 mM NaCl and 50 μM H_2_O_2_ and after the addition of 10 mM, 20 mM, 30 mM, 40 mM and 50 mM ASA in 50 mM acetate buffer, pH 5, 0 at 25 °C. The myeloperoxidase concentration was 5 μg/mL (0.25 U/mL) in all reactions.

With the addition of ASA, the decrease in absorbance at 290 nm was markedly inhibited. The absorbance decreased by about 0.98 (from 1 to 0.024) after 10 min in the reaction without ASA, by about 0.74 (from 1 to 0.26) in the presence of 10 mM ASA, and by about 0.32 (from 1 to 0.68) in the presence of 20 mM ASA, while no decrease in absorbance was observed with 30 mM, 40 mM and 50 mM ASA (Figure 4a). After determining the initial speed of the individual reactions from the progress curves, the percentage inhibition of MPO activity in the reaction with MCD was determined. As shown in Figure 4b, the percentage inhibition was 99.82 ± 0.58% for 30 mM, 99.51 ± 1.73% for 40 mM and 99.89 ± 0.64% for 50 mM ASA, 79.93 ± 6.43% for 20 mM ASA and 40.04 ± 3.21% for 10 mM ASA.

### 2.4. Oxidation of 4-Aminoantipyrine by MPO in the Presence of ASA

**Figure 5:**
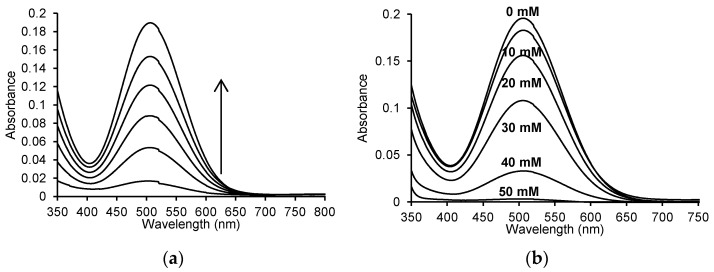
(**a**) Time spectra recorded for the reaction of 50 μM 4-aminoantipyrine, 1 mM phenol, 100 μM H_2_O_2_, and 2.5 μg/mL (0.125 U/mL) myeloperoxidase in 50 mM sodium phosphate buffer, pH 7.4 at 25 °C; (**b**) spectra after 10 min for the enzymatic oxidation reaction of 50 μM 4-aminoantipyrine and 1 mM phenol with 100 μM H_2_O_2_ without ASA and in the presence of 10 mM, 20 mM, 30 mM, 40 mM and 50 mM ASA in 50 mM sodium phosphate buffer, pH 7.4 at 25 °C.

One reaction in which MPO activity can be measured is the reaction with 4-aminoantipyrine. This reaction proceeded over time, and we studied its progress using a Jasco V-650 UV-Vis spectrophotometer. Figure 5a shows the UV-Vis spectra of the reaction progress in the range of 350 to 800 nm, recorded every 2 min for 10 min. Recording the spectra of this reaction allowed the identification of the wavelength at which the resulting product showed the highest absorbance. The oxidised form of 4-aminoantipyrine shows the maximum absorbance at a wavelength of about 505 nm. The addition of various concentrations of ASA to the mixture resulted in a decrease in the absorbance at 505 nm (Figure 5b).

When recording the UV-Vis spectra with and without the addition of ASA in the reaction mixture, visible differences in absorbance were observed. The maximum absorbance at 505 nm was 0.195 ± 0.001% for the enzymatic reaction without the addition of ASA. A decrease in the absorbance value was observed after adding indicated concentrations of ASA, corresponding to 0.012 for 10 mM ASA (A_505 nm_ = 0.183 ± 0.001%), 0.039 for 20 mM ASA (A_505 nm_ = 0.156 ± 0.004%), 0.087 for 30 mM ASA (A_505 nm_ = 0.108 ± 0.002%), 0.163 for 40 mM ASA (A_505 nm_ = 0.033 ± 0.0005%) and 0.192 for 50 mM ASA (A_505 nm_ = 0.003 ± 0.0007%).

**Figure 6:**
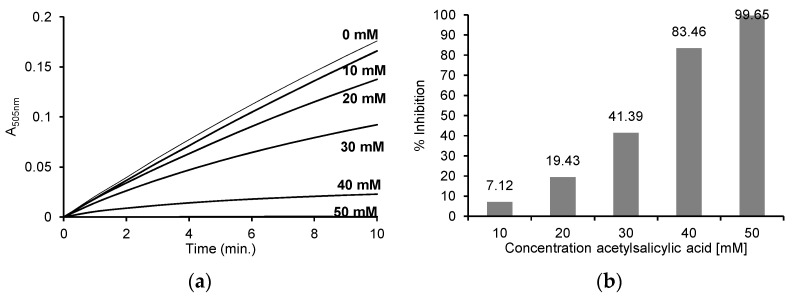
(**a**) Reaction progress curves at 505 nm for the enzymatic oxidation of 50 μM 4-aminoantipyrine, 1 mM phenol, 100 μM H_2_O_2_ without ASA and in the presence of 10 mM, 20 mM, 30 mM, 40 mM and 50 mM ASA in 50 mM sodium phosphate buffer, pH 7.4 at 25 °C; (**b**) the % inhibition for individual concentrations of ASA after enzymatic oxidation of 50 μM 4-aminoantipyrine, 1 mM phenol, 100 μM H_2_O_2_ and after the addition of 10 mM, 20 mM, 30 mM, 40 mM and 50 mM ASA in 50 mM sodium phosphate buffer, pH 7.4 at 25 °C. The myeloperoxidase concentration was 2.5 μg/mL (0.125 U/mL) in all reactions.

Based on the recorded reaction progress curves at 505 nm for the different concentrations of ASA (Figure 6a), the initial speed and % inhibition of each reaction were determined (Figure 6b). The obtained results show that the reaction rate was inhibited by 7.12 ± 6.39% in the case of 10 mM ASA and by 19.43 ± 3.51% in the case of 20 mM ASA. The presence of higher concentrations of ASA (30, 40 and 50 mM) resulted in the significant inhibition of this reaction, by 41.39 ± 1.89%, 83.46 ± 1.91% and 99.65 ± 0.02%, respectively.

## 3. Discussion

MPO is a protein that plays a key role in the non-specific antibacterial defence system [14]. This enzyme is released in the process of phagocytosis and catalyses oxidation reactions of compounds such as halides or thiocyanates in the presence of hydrogen peroxide, producing the appropriate acids, which are strong and effective bactericidal substances [1,2]. Whilst the oxidants produced by MPO play an important role in the destruction of microorganisms and the inactivation of viruses, there is growing evidence that the increased activity of this enzyme and the accumulation of peroxidase-induced oxidative damage contribute to the development of many diseases. The MPO-H_2_O_2_-Cl^−^ system and hypochlorous acid enhance the oxidation of low-density lipoproteins (LDL), which may contribute to the development of atherosclerosis. MPO effectively reverses the cardioprotective effect of high-density lipoproteins (HDL), which contributes to the development of cardiovascular diseases. The correlation of active MPO with the severity of cystic fibrosis has also been proven. Meanwhile, oxidants generated by peroxidases including HClO contribute to lung damage and dysfunction. It has been shown that MPO contributes directly or indirectly to many other diseases, but the most dangerous is cancer. MPO found in the lungs may contribute to lung cancer by activating harmful substances, including aromatic amines. Studies have shown elevated levels of MPO and oxidative stress markers in colonic lesions, demonstrating that MPO-derived oxidants produced by activated neutrophils play a role in carcinogenesis. Increased levels of MPO are also associated with many other types of cancer, e.g., leukaemia [2] and gynaecological cancers [6], including ovarian cancer [5,15,16,17,18].

MPO is necessary for the release of extracellular neutrophil (NET) traps, which have been associated with cancer progression in several preclinical models [19]. NET can both promote and inhibit tumour progression. NET release inhibited metastasis and was cytotoxic to melanoma cells in vitro. In contrast, NETs promoted metastasis in breast cancer models, indicating that perhaps the outcome of NET activity is model dependent and perhaps canonical vs. non-canonical MPO function [20].

The effect of MPO on carcinogenesis is probably multidirectional and involves numerous signal transduction pathways. The administration of exogenous MPO in a mouse breast cancer model stimulated a significant increase in tumour size compared to control animals [21]. The observed effect of MPO on tumour growth was most likely due to its augmenting pro-tumourigenic collagen production, angiogenesis and interaction with the surrounding microenvironment. It was also observed that the administration of exogenous MPO increased lung metastasis in treated animals, which may prove the protumour properties of MPO. In contrast, MPO-derived hypochlorous acid (HOCl) was suggested to protect against tumourigenesis by inhibiting NF-κβ [22].

Ovarian cancer is a heterogeneous disease with the highest mortality and the worst prognosis among the gynaecological malignancies [23,24]. Every year, around 230,000 women worldwide are diagnosed with ovarian cancer and more than 150,000 die from the disease. This is largely due to the fact that it does not give specific symptoms until the late stage of the disease [25,26]. The standard treatment for ovarian cancer involves either primary offload surgery followed by platinum-based chemotherapy, or neoadjuvant chemotherapy followed by interval offload surgery and additional postoperative chemotherapy. However, the therapeutic approach is only effective in a small number of patients, and the prognosis for ovarian cancer remains poor, with an overall 5-year survival rate ranging from 30% to 50% [23]. For this reason, there is a growing desire to use preventive methods in cancer treatment and to develop economical and effective chemopreventive agents, such as ASA, which is a representative NSAID [27]. The efficacy of aspirin may result from its ability to inhibit COX enzymes by the irreversible acylation of serine residues, which in turn leads to the inhibition of prostaglandin synthesis, which causes inflammation, fever, pain and swelling [10].

Recently, Harper AK et al. showed that ovarian carcinoma (OC) cells and tissues express MPO, which plays a key role in immune surveillance and inflammation. MPO is colocalized with inducible nitric oxide synthase (iNOS), a key pro-oxidant enzyme, and plays a key role in regulating apoptosis in OC cells. Myeloid cells express MPO in a dimeric form, while the authors found the unique expression of only the monomeric form of MPO in OC cells, tissues and blood of an ovarian cancer patients. They identified a cell membrane receptor, integrin αV/β1, which is present in both platinum-sensitive and chemo-resistant OCs with significantly higher expression in platinum-resistant OCs. Additionally, this group of authors showed that monoclonal antibodies against αV/β1 integrin induced cytotoxicity in OC cells, but not in normal cells. The discovered cytotoxicity mechanism of αV/β1 antibodies most likely results from conformational changes in αV/β1 integrin, which prevent monomeric binding of MPO with αV/β1 integrin, inhibiting MPO activation, leading to increased apoptosis. This mechanism may be the basis for the development of new targeted therapies for ovarian cancer [28].

Non-steroidal anti-inflammatory drugs are one of the most commonly used drugs by patients. Their analgesic, antipyretic and anti-inflammatory properties are widely known. Many experimental and epidemiological data confirm that NSAIDs also have clinically significant anticancer properties. The first reports on the possibility of using these compounds in cancer therapy appeared in the 1980s. For the first time, in 1983, Pollard and Lucket showed that the administration of NSAIDs for several weeks reduced the number and size of colorectal tumours in rats [29]. Similar results were obtained by authors studying other non-steroidal anti-inflammatory drugs in similar systems: piroxicam [30], sulindac [31], ibuprofen [32] and Craven and DeRubertis confirmed these reports using ASA [32]. In the presented studies, the inhibitory effect of NSAIDs on the development of neoplastic tissue was dependent on the dose of the drug used and the moment of its action. In the work of Moorghen et al., it was shown that sulindac inhibited the formation of chemically induced colon cancer in mice only when it was administered throughout the experiment, i.e., from the moment of using the carcinogen [33]. In the case of piroxicam, such a strong dependence on the time of administration was not observed. This drug showed a protective effect even in the 13th week after the use of a carcinogen, reducing the incidence and number of intestinal tumours in a dose-dependent manner [30]. After the publication of animal studies, clinical trials using NSAIDs began to be conducted. The first study was performed by Waddell and Loughry in four patients with familiar adenomatous polyps using sulindac [34]. In 1988, it was observed that patients regularly taking aspirin were less likely to develop and die from colon cancer [35]. In the following years, many controlled studies were carried out. A large number of studies focus on the anticancer effect of NSAIDs in various models of colorectal cancer, both live and prospective, most of which confirmed the prophylactic effect of aspirin and NSAIDs in colorectal cancer, but observations were subsequently made on people with various types of cancer, and the anti-cancer properties of aspirin were noted in the case of gastrointestinal cancer, gastric cancer, colorectal cancer [36,37,38], lung cancer [39], skin cancer [40], bladder cancer [41] and breast cancer [39,42]. Long-term aspirin intake has been shown to reduce the risk and improve survival in patients with colon, breast, prostate and endometrial cancer [43,44,45,46,47,48,49,50], and the regular use of this NSAID is associated with a reduced risk of ovarian cancer [43,51,52]. Aspirin can inhibit tumour progression and increase the sensitivity of OC cells to platinum salts by p53 acetylation and also the activation of p53 target genes that regulate tumour migration, proliferation and chemoresistance [10].

In 2023, Hurwitz et al. presented a study that analyzed data from 4476 patients with nonmucinous ovarian cancer and 6659 participants from the control group. Frequent use of aspirin in the first group was reported by 575 women, while in the control group, it was used by 1030 people. The analysis found an association between frequent aspirin use and a lower risk of ovarian cancer in women who were genetically predisposed to the disease. As reported, frequent use of aspirin was associated with a 13% reduction in the risk of nonmucinous ovarian cancer. The risk reduction was most significant for high-grade serous and endometrioid tumours [53].

There have been initial preclinical reports on the role of selective MPO inhibitors, verdiperstat and AZD5904 in supporting the effects of cancer immunotherapy. The combination of verdiperstat or AZD5904 with immune checkpoint inhibitors (ICIs) significantly improved long-term survivors in melanoma YUMM3.3 tumour-bearing adult WT animals [28].

Since many studies have shown an increase in the expression of MPO in ovarian cancer tissues, the study of compounds that could potentially inhibit the activity of this enzyme is very important. In our work, we have presented the effects of one NSAID, ASA, and showed that this compound inhibits the activity of MPO.

## 4. Materials and Methods

### 4.1. Chemicals and Reagents

MPO from human leukocytes (≥50 units/mg protein), 4-aminoantipyrine and *o*-dianisidine dihydrochloride were purchased from Merck (Saint Louis, MO, USA). 2-Chloro-5,5-dimethyl-1,3-cyclohexanedione (95%) was purchased from Thermo Fisher Scientific (Waltham, MA, USA). Monochlorodimedone (MCD) was purchased from Alfa Aesar (Haverhill, MA, USA). Hydrogen peroxide, phenol, buffer components and solvents were purchased from Avantor Performance Materials (Gliwice, Poland). *ASA* was purchased from WarChem (Warsaw, Poland). All reagents were of analytical grade.

### 4.2. Spectrophotometric Measurements

*O*-dianisidine dihydrochloride, 4-aminoantipyrine and phenol were dissolved in 50 mM sodium phosphate buffer, pH 7.4. Hydrogen peroxide and *ASA* were dissolved in 50 mM sodium phosphate buffer, pH 7.4 or 50 mM acetate buffer, pH 5.0. 2-Chloro-5,5-dimethyl-1,3-cyclohexanedione and sodium chloride were dissolved in 50 mM acetate buffer, pH 5.0. The MPO from human leukocytes was prepared in redistilled water. Measurements were performed using a Jasco V-650 UV-Vis (Jasco International Co., LTD, Tokyo, Japan) spectrophotometer with 1 cm-pathlength quartz cuvettes (Hellma Suprasil, Hellma GmbH & Co. KG, Müllheim, Germany). The reactions were carried out in 1 mL of 50 mM sodium phosphate buffer, pH 7.4 or 50 mM acetate buffer, pH 5.0 at 25 °C. All experiments were performed at least in triplicate.

### 4.3. The Effect of ASA on the Peroxidase Activity of MPO

The reactions were carried out at 25 °C in 1 mL of 50 mM sodium phosphate buffer, pH 7.4, containing 130 μM *o*-dianisidine dihydrochloride, 60 μM hydrogen peroxide and 5 μg/mL (0.25 U/mL) MPO. Acetylsalicylic acid was used at 10, 20, 30, 40 or 50 mM concentration, and the absorbance was measured at 460 nm for 10 min. The reference cuvette contained buffer alone. Additionally, the same reactions were carried out with 10, 20, 30, 40 or 50 mM ASA, and spectra were recorded from 200 to 800 nm immediately after mixing the reagents and then at 1 min intervals for 10 min. The reactions were started by the addition of the enzyme.

### 4.4. The Effect of ASA on the Chlorinating Activity of MPO

The reactions were carried out at 25 °C in 1 mL of 50 mM sodium acetate buffer, pH 5.0, containing 50 μM monochlorodimedone (MCD), 100 mM sodium chloride, 50 μM hydrogen peroxide and 5 μg/mL (0.25 U/mL) MPO. ASA was used at 0.25, 0.5, 1.0, 2.0, 10.0, 20, 30, 40 or 50 mM concentrations, and the absorbance was measured at 290 nm for 10 min. The reference cuvette contained buffer alone. Additionally, the same reactions were carried out with 0.25, 0.5, 1.0, 2.0, 10, 20, 30, 40 or 50 mM ASA, and spectra were recorded from 200 to 800 nm immediately after mixing the reagents and then at 1 min intervals for 10 min. The reactions were started by the addition of the enzyme.

### 4.5. The Effect of ASA on MPO Activity Using 4-Aminoantipyrine as a Hydrogen Donor

The reactions were carried out at 25 °C in 1 mL of 50 mM phosphate buffer, pH 7.4, containing 50 μM 4-aminoantipyrine, 1 mM phenol, 100 μM hydrogen peroxide and 2.5 μg/mL (0.125 U/mL) MPO. ASA was used at 10, 20, 30, 40 or 50 mM concentrations, and the absorbance was measured at 505 nm for 10 min. The reference cuvette contained buffer alone. Additionally, the same reactions were carried out with 10, 20, 30, 40 or 50 mM ASA, and spectra were recorded from 200 to 800 nm immediately after mixing the reagents and then at 1 min intervals for 10 min. The reactions were started by the addition of the enzyme.

## 5. Conclusions

Acetylsalicylic acid as a representative of non-steroidal anti-inflammatory drugs has been attracting interest in the world of science for years. Its effect is very often associated with a better prognosis of cancer patients’ survival. In our studies, we have shown that ASA significantly affects the activity of myeloperoxidase. It inhibits both peroxidase and chlorination activity.

## Data Availability

The data presented in this study are available on request from the corresponding author.
